# Influence of delivery mode on maternal mental status one month after delivery at a perinatal center in Japan: A cross-sectional study

**DOI:** 10.12688/f1000research.20677.1

**Published:** 2019-10-15

**Authors:** Shunji Suzuki

**Affiliations:** 1 Japanese Red Cross Katsushika Maternity Hospital, Department of Obstetrics and Gynecology, 5-11-12 Tateishi, Katsushika-ku, Tokyo, 124-0012, Japan

**Keywords:** maternal mental status, elective cesarean section, birth-review

## Abstract

**Background:** Maternal mental status has been thought to be affected by the delivery modes. We examined the influence of delivery modes on the mental status of women who delivered at our institute in Japan.

**Methods:** Data were collected from the medical charts of 645 primiparous women without a history of mental disorders who delivered singleton babies and underwent a 1-month postpartum check-up at our institute from September 2018 to June 2019. The maternal mental status was examined based on the scores of the Edinburgh Postnatal Depression Scale (EPDS) and the Mother-Infant Bonding Scale (MIBS).

**Results:** The rate of high scores of the EPDS and the MIBS in women choosing elective cesarean section were higher than in women with vaginal delivery and emergency cesarean section.

**Conclusion:** A fulfilling birth-plan and birth-review may also be necessary for women choosing elective cesarean section.

## Introduction

Maternal mental status has been thought to be affected by the delivery modes because childbirth is an important event for both the mother and child, and it influences early mother-infant interaction ^1, 2^. In this study, we examined the influence of delivery modes on the mental status of women who delivered at our institute in Japan.

## Methods

### Ethical issues

The protocol for this study was approved by the Ethics Committee of the Japanese Red Cross Katsushika Maternity Hospital. In addition, informed consent concerning analysis from a retrospective database was obtained from all subjects. In our institute, cesarean section is not performed without medical indication because cesarean section on maternal request for pain relief has not been generally recognized in Japan.

### Data collection

Data were collected from the medical charts of 645 primiparous women without a history of mental disorders who delivered singleton babies and underwent a 1-month postpartum check-up at our institute from September 2018 to June 2019. Of the 645 primiparous women, 389 women (60.3%) had vaginal deliveries, 80 (12.4%) had elective cesarean deliveries, and 176 (27.3%) had emergent cesarean deliveries. In this study, demographic data included maternal age. The maternal mental status was examined based on the scores of the Edinburgh Postnatal Depression Scale (EPDS) and the Mother-Infant Bonding Scale (MIBS), and the time required for psychiatric counseling by our midwives. Women with the EPDS ≥ 9 points, those with the MIBS ≥ 3 points, and the time required for psychiatric counseling ≥ 25 minutes were diagnosed with mental problems.

### Data analysis

Data are presented as mean ± SD or number (%). SPSS Statistics software version 20 (IBM Corp., Armonk, NY, USA) was used for statistical analyses. For statistical analysis, the *Χ ^2^* test for categorical variables and the Student’s *t*-test for continuous variables were used. Differences with *p* < 0.05 were considered significant.

## Results

Table 1 shows the clinical description of primiparous women and the results of mental problems. The rates of high scores of the EPDS and the MIBS were higher in the emergency cesarean group than vaginal delivery group; in addition, the rate of high scores of the EPDS and the MIBS and a long time for psychiatric counseling in women choosing elective cesarean section were higher than in women with vaginal delivery and emergency cesarean section, as shown in Table 1.

**Table 1.** Clinical description and the results of mental problems in nulliparous women who delivered singleton babies at one month after delivery.

Variable	Vaginal delivery	Elective cesarean delivery	Emergency cesarean delivery
Number	387	80	176
EPDS			
Average (points)	5.1±4.0	5.8±4.6 *	5.4±3.5
≥9 points	23 (6.0)	22 (27.5) * #	21 (11.9) *
MIBS			
Average (points)	1.8±1.8	2.7±2.9 *	2.4±2.4 *
≥3 points	33 (8.5)	21 (26.3) *	31 (17.6) *
Interview time			
Average (minutes)	15.4±9.0	18.5±8.0 *	14.0±6.2
≥25 minutes	31 (8.0)	12 (15.0) * #	8 (4.5)

Data are presented as mean ± SD or number (%). EPDS, Edinburgh Postnatal Depression Scale; MIBS, Mother-Infant Bonding Scale. * *P* vs. vaginal delivery group. # *P* vs. vaginal and emergency cesarean delivery groups.

## Discussion

This may be the first report to indicating that women received elective cesarean section are more prone to have mental problems. Although we predicted that the highest frequency of mental problems would be in the emergent cesarean delivery group, the women choosing elective cesarean delivery actually had the most mental problems. The reason for the results is not clear; however, based on the records of psychiatric counseling, it may be because there was no birth-plan or birth-review for women scheduled for elective cesarean delivery. In our institute, a birth-plan has been carried out for all pregnant women scheduled for vaginal delivery, and a birth-review that takes a long time during hospitalization has been performed especially for mothers undergoing emergency caesarean section in order to recover from the trauma of the sudden departure from normal labor ^3^. This is because a birth-review is one of the concrete measures to learn about the ‘bruising’ of labor and promptly affirm the experience of delivery ^3, 4^. A mother’s thought during birth-review about the experience of childbirth has been suggested to help express feelings of embarrassment and provide an opportunity to reconstruct the facts. On the other hand, pregnant woman scheduled to undergo elective cesarean section are given an explanation and birth-review of cesarean section solely from a surgical perspective. The absence of an adequate birth-plan or birth-review may lead to mental problems in postpartum women who receive elective cesarean section.

We understand the small sample size for statistical analyses as one of serious limitations in this study. However, a fulfilling birth-plan and birth-review may also be necessary for women choosing elective cesarean section.

## Data availability

### Underlying data

Figshare: delivery mode and maternal mental status. https://doi.org/10.6084/m9.figshare.9956690.v1 ^5^.

This project contains data on the delivery method, EPDS and MIBS scores and counselling time for each participant.

Data are available under the terms of the Creative Commons Zero "No rights reserved" data waiver (CC0 1.0 Public domain dedication).
