# Supplementary material for: Relation between delivery mode and maternal mental status one month after delivery at a perinatal center in Japan: A cross-sectional study
Source: F1000Res. 2022 Oct 27;8:1755. Originally published 2019 Oct 15. [Version 4] doi: 10.12688/f1000research.20677.4 (PMC9614284; doi:10.12688/f1000research.20677.4)
Supplement: Peer review history

# Peer review: Reviewer response for version 4

**DOI:** 10.5256/f1000research.140176.r154375

**Published:** 2022-10-27

**Authors:** Jacopo Lenzi

**Affiliations:** 1 Department of Biomedical and Neuromotor Sciences, Alma Mater Studiorum - University di Bologna, Bologna, Italy

I have no further comments to make.

Is the work clearly and accurately presented and does it cite the current literature?

Partly

If applicable, is the statistical analysis and its interpretation appropriate?

No

Are all the source data underlying the results available to ensure full reproducibility?

Yes

Is the study design appropriate and is the work technically sound?

Partly

Are the conclusions drawn adequately supported by the results?

Partly

Are sufficient details of methods and analysis provided to allow replication by others?

Yes

Reviewer Expertise:

NA

I confirm that I have read this submission and believe that I have an appropriate level of expertise to confirm that it is of an acceptable scientific standard.

# Peer review: Reviewer response for version 3

**DOI:** 10.5256/f1000research.28688.r69151

**Published:** 2020-08-18

**Authors:** Jacopo Lenzi

**Affiliations:** 1 Department of Biomedical and Neuromotor Sciences, Alma Mater Studiorum - University di Bologna, Bologna, Italy

S. Suzuki addressed all the concerns I raised except one. Looking at the new version of the work, it seems to me that he reviewed the table header and footnotes, but not the table itself. In this way, the header does not match with the table (no estimate is provided for the whole sample). He should update the frame and content of the table following my previous comment, where columns are separated by '\' and rows are separated by '|' (I know it is tricky to read, but I could not paste the Word table in the online form).

 If the author addresses this point, his work can be accepted without any further changes.

Is the work clearly and accurately presented and does it cite the current literature?

Partly

If applicable, is the statistical analysis and its interpretation appropriate?

No

Are all the source data underlying the results available to ensure full reproducibility?

Yes

Is the study design appropriate and is the work technically sound?

Partly

Are the conclusions drawn adequately supported by the results?

Partly

Are sufficient details of methods and analysis provided to allow replication by others?

Yes

Reviewer Expertise:

NA

I confirm that I have read this submission and believe that I have an appropriate level of expertise to confirm that it is of an acceptable scientific standard, however I have significant reservations, as outlined above.

# Peer review: Reviewer response for version 2

**DOI:** 10.5256/f1000research.27256.r68783

**Published:** 2020-08-06

**Authors:** Jacopo Lenzi

**Affiliations:** 1 Department of Biomedical and Neuromotor Sciences, Alma Mater Studiorum - University di Bologna, Bologna, Italy

The work by Shunji Suzuki analyzes the association between method of delivery and maternal mental status after one month. He found that elective C-sections are associated with higher levels of mental distress, and argued that this result might have to do with the absence of birth-plans and birth-reviews for women undergoing elective CS. Here are my comments:

1. I would not talk about “influence”, which implies causality. Please consider using “association” or “relationship” instead.
2. Are you sure that your sample size is 645? According to the data presented in Table 1 and uploaded to Figshare, the women enrolled in the study are 643.
3. No justification is provided for the cut-offs presented at the end of the Data Collection section. For instance, you used an EPDS cut-off of 9, but more commonly adopted thresholds are 10 (minor depression) and 13 (major depression) (Fellmeth G, et al. *J Affect Disord* 2019;251:8-14). Please provide adequate references or update your cut-offs.
4. Table 1 and the data set you shared do not match. After analyzing the data uploaded to Figshare, I found very different results (see below). Please check consistency and review the entire Results section, if necessary. Please also consider adding a column for the entire sample, as I did here. Statistical significance should also be described differently in the table footnotes.
**Table 1.** One-month clinical evaluation for women who delivered a singleton baby between September 2018 and June 2019, overall and by method of delivery.
  ---
  Variable \ All ( *n *= 643) \ Vaginal delivery ( *n *= 387) \ Elective CS ( *n *= 80) \ Urgent CS ( *n *= 176) |
  EPDS score |
  Mean ± SD \ 3.7 ± 3.4 \ 3.2 ± 2.7 \ 5.5 ± 4.9*# \ 4.0 ± 3.8* |
  ≥9, *n* (%) \ 66 (10.3) \ 23 (5.9) \ 22 (27.5)*# \ 21 (11.9)* |
  MIBS score |
  Mean ± SD \ 1.3 ± 2.1 \ 1.1 ± 2.1 \ 1.9 ± 2.4*# \ 1.3 ± 2.1 |
  ≥3, *n* (%) \ 86 (13.4) \ 33 (8.5) \ 22 (27.5)*# \ 31 (17.6)* |
  Interview time (minutes) |
  Mean ± SD \ 12.8 ± 7.3 \ 12.9 ± 7.8 \ 14.1 ± 8.3 \ 11.8 ± 5.3 |
  ≥25, *n* (%) \ 52 (8.1) \ 32 (8.3) \ 12 (15.0)# \ 8 (4.5) |
  ---
*CS*, cesarean section; *EPDS*, Edinburgh Postnatal Depression Scale; *MIBS*, Mother-Infant Bonding Scale.
  * *p* <0.05 (elective CS vs. vaginal delivery).
  # *p* <0.05 (elective CS vs. vaginal delivery + urgent CS).
5. What about maternal age? Was it similar across the three groups under study? If so, the association between method of delivery and outcomes should be adjusted for age.
6. What about the external validity (generalizability) of the study? The Katsushika Maternity Hospital does not have any “birth-path” for women undergoing elective C-section—is this a common or uncommon situation?

Is the work clearly and accurately presented and does it cite the current literature?

Partly

If applicable, is the statistical analysis and its interpretation appropriate?

No

Are all the source data underlying the results available to ensure full reproducibility?

Yes

Is the study design appropriate and is the work technically sound?

Partly

Are the conclusions drawn adequately supported by the results?

Partly

Are sufficient details of methods and analysis provided to allow replication by others?

Yes

Reviewer Expertise:

Medical statistics, Epidemiology

I confirm that I have read this submission and believe that I have an appropriate level of expertise to confirm that it is of an acceptable scientific standard, however I have significant reservations, as outlined above.

# Peer review: Reviewer response for version 2

**DOI:** 10.5256/f1000research.27256.r64608

**Published:** 2020-06-16

**Authors:** Tuğba Kınay

**Affiliations:** 1 Department of Gynaecology, Etlik Zübeyde Hanım Education and Research Hospital, University of Health Sciences, Ankara, Turkey

I reviewed the revised manuscript. The author revised the manuscript based on my comment. I think that the paper is suitable for indexing in its current form.

Is the work clearly and accurately presented and does it cite the current literature?

Yes

If applicable, is the statistical analysis and its interpretation appropriate?

Yes

Are all the source data underlying the results available to ensure full reproducibility?

Yes

Is the study design appropriate and is the work technically sound?

Yes

Are the conclusions drawn adequately supported by the results?

No

Are sufficient details of methods and analysis provided to allow replication by others?

Yes

Reviewer Expertise:

Obstetrics and gynecology

I confirm that I have read this submission and believe that I have an appropriate level of expertise to confirm that it is of an acceptable scientific standard.

# Peer review: Reviewer response for version 1

**DOI:** 10.5256/f1000research.22741.r63674

**Published:** 2020-06-08

**Authors:** Tuğba Kınay

**Affiliations:** 1 Department of Gynaecology, Etlik Zübeyde Hanım Education and Research Hospital, University of Health Sciences, Ankara, Turkey

Thank you for the opportunity to evaluate this report. The paper is about the effect of  delivery mode on the postpartum mental status of women. The results of the study indicate that the women who underwent elective cesarean delivery had higher scores of the EPDS and MIBS than women with vaginal delivery and emergent cesarean delivery.  I have the following comment:

 In discussion section,  the author state that – "We understand the small sample size for statistical analyses as one of serious limitations in this study. However, a fulfilling birth-plan and birth-review may also be necessary for women choosing elective cesarean section." I don’t think that the current study based on its methodology can support that statement .The conclusion paragraph including main outcomes of the study should be rewritten.

Is the work clearly and accurately presented and does it cite the current literature?

Yes

If applicable, is the statistical analysis and its interpretation appropriate?

Yes

Are all the source data underlying the results available to ensure full reproducibility?

Yes

Is the study design appropriate and is the work technically sound?

Yes

Are the conclusions drawn adequately supported by the results?

No

Are sufficient details of methods and analysis provided to allow replication by others?

Yes

Reviewer Expertise:

obstetrics and gynecology

I confirm that I have read this submission and believe that I have an appropriate level of expertise to confirm that it is of an acceptable scientific standard, however I have significant reservations, as outlined above.
